# Respiratory Support Techniques for COVID-19-Related ARDS in a Sub-Saharan African Country: A Multicenter Observational Study

**DOI:** 10.1016/j.chest.2023.01.039

**Published:** 2023-02-10

**Authors:** Arthur Kwizera, Daphne Kabatooro, Patience Atumanya, Janat Tumukunde, Joyce Kalungi, Arthur Kavuma Mwanje, Daniel Obua, Peter Agaba, Cornelius Sendagire, Jane Nakibuuka, Darius Owachi, Martin W. Dünser, Anne Alenyo-Ngabirano, Charles Olaro, Henry Kyobe-Bosa, Bruce J. Kirenga, Lydia Nakiyingi, Noah Kiwanuka, David Patrick Kateete, Moses Joloba, Nelson Sewankambo, Charlotte Summers

**Affiliations:** a Department of Anaesthesia and Critical Care, Makerere University College of Health Sciences, Kampala, Uganda; b Department of Medicine, Makerere University College of Health Sciences, Kampala, Uganda; c School of Public Health, Makerere University College of Health Sciences, Kampala, Uganda; d Department of Immunology and Molecular Biology, School of Biomedical Sciences, Makerere University College of Health Sciences, Kampala, Uganda; e Uganda Heart Institute, Kampala, Uganda; f Department of Medicine and Intensive Care Unit, Mulago National Referral Hospital, Kampala, Uganda; g Kiruddu National Referral Hospital, Kampala, Uganda; h Ministry of Health, Kampala, Uganda; i Uganda Peoples Defence Forces, Kampala, Uganda; j Makerere University Lung Institute, Kampala, Uganda; k Department of Anaesthesiology and Intensive Care Medicine, Kepler University Hospital and Johannes Kepler University Linz, Linz, Austria; l Kellogg College, University of Oxford, Oxford, England; m Department of Medicine, University of Cambridge, Cambridge, England

**Keywords:** ARDS, COVID-19, respiratory support technique, sub-Saharan Africa, Uganda

## Abstract

**Background:**

Limited data from low-income countries report on respiratory support techniques in COVID-19-associated ARDS.

**Research Question:**

Which respiratory support techniques are used in patients with COVID-19-associated ARDS in Uganda?

**Study Design and Methods:**

A multicenter, prospective, observational study was conducted at 13 Ugandan hospitals during the pandemic and included adults with COVID-19-associated ARDS. Patient characteristics, clinical and laboratory data, initial and most advanced respiratory support techniques, and 28-day mortality were recorded. Standard tests, log-rank tests, and logistic regression analyses were used for statistical analyses.

**Results:**

Four hundred ninety-nine patients with COVID-19-associated ARDS (mild, n = 137; moderate, n = 247; and severe, n = 115) were included (ICU admission, 38.9%). Standard oxygen therapy (SOX), high-flow nasal oxygen (HFNO), CPAP, noninvasive ventilation (NIV), and invasive mechanical ventilation (IMV) was used as the first-line (most advanced) respiratory support technique in 37.3% (35.3%), 10% (9.4%), 11.6% (4.8%), 23.4% (14.4%), and 17.6% (36.6%) of patients, respectively. The first-line respiratory support technique was escalated in 19.8% of patients. Twenty-eight-day mortality was 51.9% (mild ARDS, 13.1%; moderate ARDS, 62.3%; severe ARDS, 75.7%; *P* < .001) and was associated with respiratory support techniques as follows: SOX, 19.9%; HFNO, 31.9%; CPAP, 58.3%; NIV 61.1%; and IMV, 83.9% (*P* < .001). Proning was used in 79 patients (15.8%; 59 of 79 awake) and was associated with lower mortality (40.5% vs 54%; *P* = .03). The oxygen saturation to Fio_2_ ratio (OR, 0.99; 95% CI, 0.98-0.99; *P < .*001) and respiratory rate (OR, 1.07; 95% CI, 1.03-1.12; *P = .*002) at admission and NIV (OR, 6.31; 95% CI, 2.29-17.37; *P < .*001) or IMV (OR, 8.08; 95% CI, 3.52-18.57; *P < .*001) use were independent risk factors for death.

**Interpretation:**

SOX, HFNO, CPAP, NIV, and IMV were used as respiratory support techniques in patients with COVID-19-associated ARDS in Uganda. Although these data are observational, they suggest that the use of SOX and HFNO therapy as well as awake proning are associated with a lower mortality resulting from COVID-19-associated ARDS in a resource-limited setting.

FOR EDITORIAL COMMENT, SEE PAGE 275

**Take-home Points**

**Study Question:** What respiratory support techniques were used among patients with COVID-19-related ARDS in a resource-constrained sub-Saharan African country?

**Results:** In 499 patients with a first diagnosis of COVID-19-related ARDS seeking treatment at 13 hospitals in Uganda, standard oxygen therapy, high-flow nasal oxygen, CPAP, noninvasive mechanical ventilation, and invasive mechanical ventilation were used as the first-line respiratory support technique in 37.3%, 10%, 11.6%, 23.4%, and 17.6% of patients, respectively. Only 38.9% of patients were admitted to the ICU, and prone positioning was used in 79 patients (15.8%). Twenty-eight-day mortality was 51.9%.

**Interpretation:** Standard oxygen therapy, high-flow nasal oxygen, CPAP, noninvasive mechanical ventilation, and invasive mechanical ventilation were used as respiratory support techniques in patients with COVID-19-associated ARDS in Uganda. The need for escalation to advanced respiratory support (especially mechanical ventilation) and resource constraints were associated with a high mortality in this population.

ARDS is the most common and lethal complication associated with SARS-CoV-2 infection.^1^ Different respiratory support techniques, including standard oxygen therapy (SOX), high-flow nasal oxygen (HFNO) therapy, CPAP, noninvasive mechanical ventilation (NIV), and invasive mechanical ventilation (IMV) have been used during the management of COVID-19-associated ARDS.^2^^,^^3^ Some recent practice guidelines have recommended that in patients with refractory hypoxemic respiratory failure despite SOX, HFNO is preferred over NIV where possible, and only if HFNO is not available and no urgent indication exists for endotracheal intubation, should a trial of NIV with close monitoring be considered.^3^^,^^4^ It is important to acknowledge that all recommendations on the indication and choice of respiratory support techniques in COVID-19-associated ARDS are based on weak scientific evidence that almost exclusively originates from high- and middle-income countries.^5^ Although pragmatic recommendations were made for low- to middle-income countries,^6^ limited data have described the practice of respiratory support used for patients with COVID-19 ARDS in these settings. This is of relevance because many low- to middle-income countries have been burdened significantly by the COVID-19 pandemic, with limited critical care capacities contributing to mortality rates for critically ill patients admitted to high-dependency units or ICUs in Africa reported to be higher than almost everywhere else in the world.^7^ When extrapolating evidence on the respiratory management of COVID-19-associated ARDS from resource-rich to resource-poor settings, it is crucial to remember that certain critical care interventions known to improve patient outcomes in high-income settings (eg, fluid resuscitation, early enteral nutrition) have been associated with harm when implemented in low- to middle-income country settings.^8^^,^^9^ In this study, we aimed to explore the clinical use and outcomes of different respiratory support techniques in patients with COVID-19-associated ARDS admitted to 13 Ugandan hospitals during the COVID-19 pandemic.

## Study Design and Methods

We conducted a multicenter prospective observational cohort study at 13 Ugandan hospitals (governmental hospitals, n = 7; private for-profit hospitals, n = 2; and nonprofit missionary hospitals, n = 4) spread throughout Uganda that served as dedicated COVID-19 centers. The study period lasted from July 1, 2020, through June 30, 2021. The study protocol was reviewed and approved by the ethics committee of the Makerere University School of Biomedical Sciences (Identifier: SBS699). Written informed consent was obtained from all study participants or their next of kin in cases where the patient was too ill to provide informed consent. Consent forms were translated into the local vernacular to aid understanding and communication. In view of the pandemic, where access to patients was limited by infection prevention controls (to cater for a period of inadequate personal protective equipment), approval for deferral of consent was obtained to enable prospective review of medical charts. Some patients (120/499) enrolled in this study were included in a simultaneous observational analysis (n = 682) evaluating the epidemiologic features and outcomes of acute hypoxemic respiratory failure in Uganda.

### Study Population and Definitions

All adult (aged ≥ 18 years) patients who required hospitalization because of suspected COVID-19-associated acute hypoxemic respiratory failure at any one of the study sites who met the criteria for ARDS at hospital admission were eligible for study enrollment. COVID-19 was diagnosed with the use of polymerase chain reaction tests to detect SARS-CoV-2 in samples obtained from nasopharyngeal swabs or tracheobronchial aspirates. In line with the modified Kigali criteria,^10^ we defined ARDS as acute hypoxemic respiratory failure (plethysmographic oxygen saturation [SpO_2_] to Fio_2_ ratio of < 316 and bilateral opacities on chest radiography or lung ultrasound) that were not explained fully by cardiac failure or fluid overload (all patients with suspected or confirmed polymerase chain reaction-positive COVID-19 underwent chest radiography as part of COVID-19 management policy). Based on the SpO_2_ to Fio_2_ ratio, ARDS was categorized as either mild (SpO_2_ to Fio_2_ ratio, 316-235), moderate (SpO_2_ to Fio_2_ ratio, 235-100), or severe (SpO_2_ to Fio_2_ ratio, < 100) at hospital admission. Patients who were in a moribund state at hospital admission (as determined by the attending physician) were excluded. The following respiratory support techniques were applied in this study population: SOX, HFNO, CPAP, NIV, and IMV. SOX was defined as oxygen application via either nasal prongs or an oxygen mask. HFNO was defined as application of heated and humidified oxygen at flow rates of ≥ 20 L/min. Whereas CPAP was defined as application of a CPAP via a tightly fitting face mask, NIV was defined as CPAP plus pressure support during inspiration. Positive pressure ventilation through an endotracheal or tracheostomy tube was defined as IMV.

### Study Setting and Clinical Management of Patients With COVID-19-Associated ARDS

In response to the COVID-19 pandemic, the Ugandan government defined a selected group of public hospitals to serve as dedicated COVID-19 treatment centers, and a national COVID-19 referral process was established (e-Fig 1). Whenever possible and where bed capacities allowed, this plan included referral of patients with COVID-19 to the dedicated COVID-19 centers and referral of severely ill patients to the national COVID-19 referral hospital at the Mulago National Referral Hospital, Kampala. All hospitals, including the COVID-19 referral centers as well as the 13 study sites, were supplied with staff training, oxygen, oxygen administration consumables to deliver SOX, and pulse oximeters for the in-hospital care of patients with COVID-19. In addition, these COVID-19 treatment centers (study sites) were provided with respiratory support devices such as HFNO and CPAP devices, as well as mechanical ventilators to deliver NIV, IMV, or both. ICUs were available in two governmental and all six nongovernmental facilities, but none initially were designated specifically for COVID-19 care. All hospitals had a high-dependency unit (HDU) for general care. However, during the pandemic surges, all study hospital ICUs and HDUs exclusively admitted COVID-19 patients. None of these hospitals offered either extracorporeal membrane oxygenation or extracorporeal CO_2_ removal therapy.

The clinical management of patients with COVID-19 at study centers was based on national guidelines published by the Ugandan Ministry of Health.^11^ Accordingly, oxygen therapy was targeted to achieve a plethysmographic SpO_2_ of ≥ 91%. Initially, oxygen was administered via nasal prongs or a face mask with a reservoir bag (SOX) and was titrated to reach the plethysmographic SpO_2_ goal. If this goal could not be achieved with SOX, therapy was escalated to an advanced respiratory support strategy as capacity allowed and as determined by the attending physician. Although HFNO, CPAP, and NIV therapy could be provided both inside and outside ICUs, IMV was provided only in an ICU setting. Patients who sought treatment at one of the study hospitals without an ICU but who required IMV were referred to an alternative health care facility with an ICU whenever possible and where bed availability allowed. All study patients received dexamethasone according to World Health Organization guidance.

### Data Collection

The following data were collected by research assistants at hospital admission in all study participants: age, sex, body weight, presence and type of any premorbid conditions, heart rate, respiratory rate, mean arterial pressure, plethysmographic SpO_2_, Fio_2_ (estimated from oxygen flow rates and oxygen application devices as suggested by the conversion tables from the Extended Prevalence of Infection in Intensive Care II study),^12^ laboratory values (hemoglobin levels, WBC and platelet counts, serum creatinine, and sodium and bilirubin concentration) wherever available, modified Sequential Organ Failure Assessment score,^13^ need for a vasopressor drug, as well as the first-line respiratory support technique that was implemented. The ratio of plethysmographic SpO_2_ or fractional inspiratory oxygen concentration to respiratory rate index was calculated as the SpO_2_ to Fio_2_ ratio divided by the respiratory rate.^14^ All patients were followed up throughout the hospital stay, and the subsequent data were documented: admission to an ICU, need for escalation of respiratory support, highest level of respiratory support required, settings when the highest respiratory support was initiated, duration of continuous respiratory support techniques (ie, HFNO and IMV), length of hospital stay, and 28-day all-cause mortality. The level of respiratory support was graded in the following increasing order of severity: SOX > HFNO > CPAP > NIV > IMV, where possible.

### Statistical Analysis

The primary objective of this study was to report the use of respiratory support techniques in patients with COVID-19-associated ARDS as well as in patients with different severity strata of COVID-19-associated ARDS. Because respiratory support was escalated in some study patients, we documented the most advanced respiratory support technique applied to each patient during the hospital stay. As secondary study objectives, we sought to determine the specific settings of respiratory support techniques and to compare them between patients with different severity strata of COVID-19-related ARDS. We further evaluated and compared the 28-day mortality associated with different respiratory support techniques and with prone positioning in patients with COVID-19-related ARDS as well as different severity strata of COVID-19-related ARDS. Finally, we determined independent risk factors for 28-day mortality in all patients with COVID-19-associated ARDS. In patients who received invasive ventilation, we sought to evaluate whether certain ventilator settings were associated independently with 28-day mortality.

Because no data regarding the use of respiratory support techniques in patients with COVID-19-associated ARDS managed in an African setting were available when the study protocol was designed, no power analysis could be undertaken, and the study was planned as an explorative analysis. We therefore chose a convenience sample of 500 participants, assuming that this number would be sufficient to address our study objectives reliably.

All data were anonymized, recorded on paper case report forms, and double-entered into an electronic database. After the cessation of data collection, the database was locked and underwent quality control checks to exclude documentation or transcription errors.

The statistical data analysis was undertaken using the PASW statistical software package (IBM Software version 20.0; IBM). Descriptive statistical methods were used to report demographic, clinical, technical, and outcome data. These parameters were compared among patients with mild, moderate, and severe ARDS using the χ^2^ test or an analysis of variance, as appropriate. Kaplan-Meier graphs were plotted for all study patients as well as study patients with mild, moderate, and severe ARDS to display cumulative survival of study participants treated with different respiratory support techniques (most advanced respiratory support technique applied during the hospital stay), as well as for study participants who underwent prone positioning, or not. Log-rank tests were used to compare 28-day all-cause mortality between groups. A binary logistic regression analysis was used to identify independent risk factors for 28-day mortality in all study participants. Variables that differed between survivors and nonsurvivors were entered as covariates into the final model. In study patients receiving IMV as the most advanced respiratory support technique, a separate binary logistic regression analysis was calculated to report the association between different ventilator settings and 28-day mortality.

For all analyses, a *P* value of < .05 was considered to indicate statistical significance. In view of the explorative nature of the study, we refrained from adjusting the α level for multiple comparisons.

## Results

**Figure 1:**
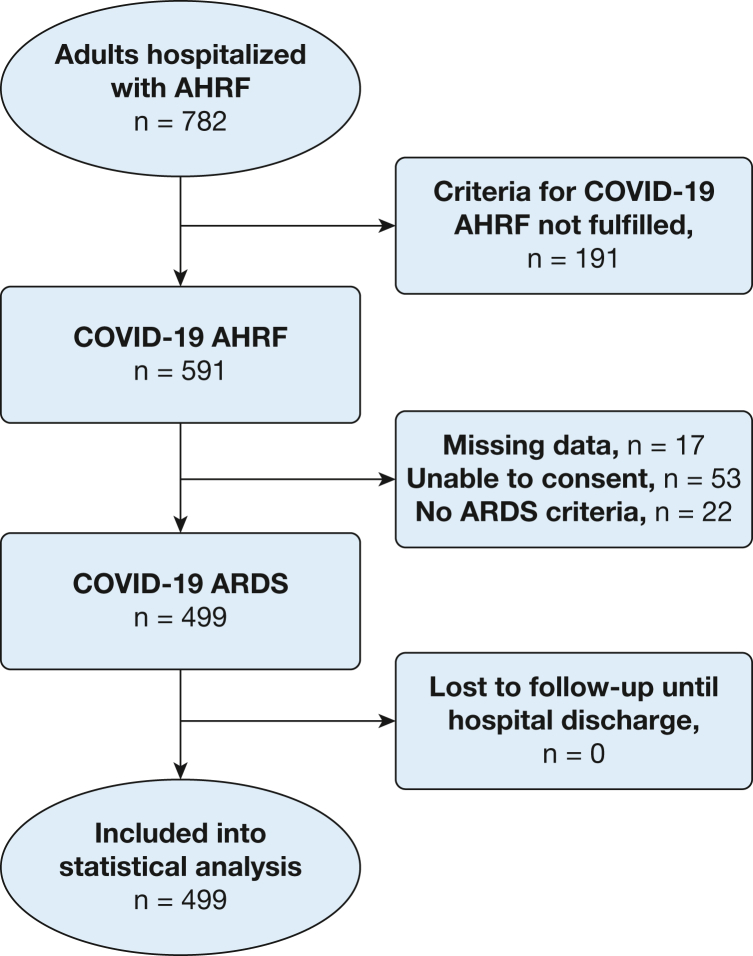
Study flow diagram. AHRF = acute hypoxemic respiratory failure.

Of 782 patients admitted to one of the study centers because of COVID-19, 499 patients met the criteria for COVID-19-related ARDS and were included in the study analyses (Fig 1, Table 1). A further breakdown is displayed in e-Table 1. Based on the SpO_2_ to Fio_2_ ratio at admission, study patients were categorized as having mild (n = 137), moderate (n = 247), or severe (n = 115) ARDS. Age, sex, prevalence of premorbid conditions, heart rate, respiratory rate, SpO_2_, SpO_2_ to Fio_2_ ratio, ratio of plethysmographic SpO_2_ or fractional inspiratory oxygen concentration to respiratory rate index, hemoglobin levels, serum bilirubin levels, the need for vasopressor support at hospital admission, rate of ICU admission, and 28-day mortality differed between study participants showing with different severity strata of COVID-19-associated ARDS.

**Table 1:** Patient Characteristics at Hospital Admission

Variable	All Study Patients	ARDS			*P* Value
		Mild	Moderate	Severe	
No.	499	137	247	115	. . .
Age, y	54 (42-65)	50 (39-59)	55 (42-65)	57 (44-70)	< .001^a^
Male sex	340 (68.1)	107 (78.1)	161 (65.2)	72 (62.6)	.012^a^
Body weight, kg	80 (70-90)	75 (63-80)	81 (73-90)	80 (70-92)	.077
Premorbid condition	249 (49.9)	52 (38)	137 (55.5)	60 (52.2)	.004^a^
Hypertension	154 (30.9)	26 (19)	89 (36)	39 (33.9)	.002^a^
Diabetes	92 (18.4)	24 (17.5)	50 (20.2)	18 (15.7)	.55
HIV	43 (8.6)	19 (13.9)	22 (8.9)	2 (1.7)	.054
Pregnancy	11 (2.2)	2 (1.5)	7 (2.8)	2 (1.7)	.63
Others	29 (5.8)	2 (1.5)	18 (7.3)	9 (7.8)	.037^a^
Vital parameters					
Heart rate, beats/min	100 (89-112)	96 (84-110)	101 (89-115)	103 (92-118)	.005^a^
Respiratory rate, breaths/min	29 (25-36)	25 (23-30)	30 (26-36)	33 (27-43)	< .001^a^
Mean arterial pressure, mm Hg	90 (80-102)	90 (78-99)	90 (79-103)	92 (85-108)	.076
SpO_2_, %	88 (80-90)	90 (87-92)	86 (79-89)	82 (68-92)	< .001^a^
SpO_2_ to Fio_2_ ratio	133 (104-215)	247 (227-274)	129 (112-151)	91 (84-96)	< .001^a^
ROX index	4.5 (3.1-7.5)	10 (8.2-11.7)	4.3 (3.4-5.2)	2.6 (2.1-3.2)	< .001^a^
Laboratory values					
Hemoglobin, g/dL	11.2 (8.7-13.5)	10 (7.6-12.4)	11.5 (8.7-13.4)	12 (8.9-15)	.032^a^
WBC count, G/L	10.4 (6.7-12.3)	8.7 (6.6-11.7)	10.6 (6.7-13.8)	10.9 (6.7-13.4)	.16
Platelet count, G/L	187 (111-282)	218 (149-286)	161 (101-248)	188 (103-273)	.054
Serum creatinine, μmol/L	90 (72-129)	90 (71-130)	91 (74-135)	88 (71-129)	.18
Serum sodium, mM	136 (131-143)	135 (132-144)	136 (130-143)	136 (131-141)	.91
Serum bilirubin, μmol/L	3.9 (1.8-8)	2 (0.8-5.8)	4.5 (2-9)	4 (2-10.7)	.044^a^
Hyponatremia	97 (19.4)	23 (16.8)	49 (19.8)	25 (21.7)	.6
Modified SOFA score	5 (4-7)	4 (3-6)	5 (4-7)	6 (4-7)	.12
Need for vasopressor	40 (8)	4 (2.9)	23 (9.3)	13 (11.3)	.029^a^
ICU or HDU admission	194 (38.9)	16 (11.7)	117 (47.4)	61 (53)	< .001^a^
Hospital LOS, d	6 (3-9)	6 (3-8)	6 (3-9)	6 (3.5-9)	.19
28-d mortality	259 (51.9)	18 (13.1)	154 (62.3)	87 (75.7)	< .001^a^

Data are presented as median (interquartile range), unless otherwise indicated. HDU = high-dependency unit; LOS = length of stay; ROX = ratio of plethysmographic oxygen saturation or fractional inspiratory oxygen concentration to respiratory rate; SOFA = sequential organ failure assessment; SpO_2_ = plethysmographic oxygen saturation.

a Significant difference among patients with mild, moderate, or severe ARDS.

### Primary Study Objective

**Figure 2:**
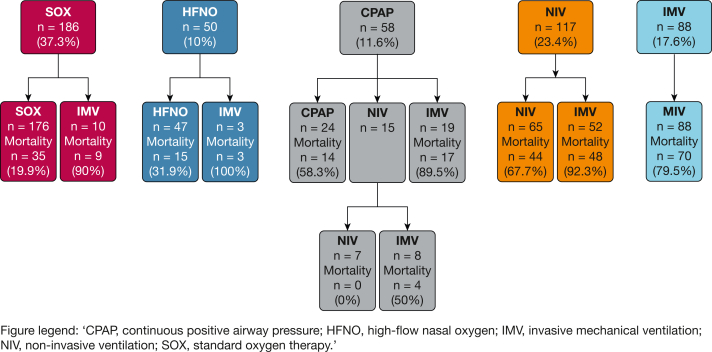
Flow diagram showing respiratory therapy techniques and escalation pathways in patients with COVID-19 ARDS. HFNO = high-flow nasal oxygen; IMV = invasive mechanical ventilation; NIV = noninvasive ventilation; SOX = standard oxygen therapy.

The use of respiratory support techniques in all study patients as well as in patients with different severity strata of COVID-19-associated ARDS are displayed in Table 2 and e-Table 1. Except for HFNO, the use of first-line and most advanced respiratory support techniques differed among patients with mild, moderate, or severe COVID-19-associated ARDS. The first-line respiratory technique was escalated in 19.8% of study patients (99/499) (Fig 2). The need to escalate the first-line respiratory technique differed among ARDS severity strata (Table 2).

**Table 2:** First-line and Most Advanced Respiratory Support Techniques

Variable	All Study Patients	ARDS			*P* Value
		Mild	Moderate	Severe	
No.	499	137	247	115	. . .
First-line respiratory support technique					
SOX	186 (37.3)	100 (73)	65 (26.3)	21 (18.3)	< .001^a^
HFNO	50 (10)	12 (8.8)	28 (11.3)	10 (8.7)	.63
CPAP	58 (11.6)	8 (5.8)	37 (15)	13 (11.3)	.028^a^
NIV	117 (23.4)	11 (8)	66 (26.7)	40 (34.8)	< .001^a^
IMV	88 (17.6)	6 (4.4)	51 (20.6)	31 (27)	< .001^a^
Escalation of first-line respiratory support	99 (19.8)	7 (5.1)	62 (25.1)	30 (26.1)	< .001^a^
Most advanced respiratory support technique					
SOX	176 (35.3)	100 (73)	57 (23.1)	19 (16.5)	< .001^a^
HFNO	47 (9.4)	12 (8.8)	26 (10.5)	9 (7.8)	.68
CPAP	24 (4.8)	2 (1.5)	12 (4.9)	10 (8.7)	.028^a^
NIV	72 (14.4)	11 (8)	45 (18.2)	16 (13.9)	.024^a^
IMV	180 (36.1)	12 (8.8)	107 (43.3)	61 (53)	< .001^a^

Data are presented as No. (%), unless otherwise indicated. HFNO = high-flow nasal oxygen; IMV = invasive mechanical ventilation; NIV = noninvasive ventilation; SOX = standard oxygen therapy.

a Significant difference among patients with mild, moderate, or severe ARDS.

### Secondary Study Objectives

Table 3 summarizes specific settings of the most advanced respiratory support techniques used in all study patients as well as in patients with different severity strata of COVID-19-associated ARDS. Except for HFNO and the absolute tidal volume in patients undergoing IMV, the settings of ventilatory support techniques did not differ among ARDS severity strata. All-cause 28-day mortality in the study population was 51.9% (259/499).

**Table 3:** Specific Settings of the Most Advanced Respiratory Support Techniques Used in the Study Population

Variable	All Study Patients	ARDS			*P* Value
		Mild	Moderate	Severe	
No.	499	137	247	115	. . .
HFNO					
Flow, L/min	40 (35-45)	40 (35-50)	40 (35-40)	45 (40-50)	.016^a^
Fio_2_, %	60 (50-60)	60 (50-60)	60 (50-80)	60 (55-60)	.16
CPAP					
PEEP, mbar	5 (5-8)	5 (5-9)	5 (5-8)	5 (5-10)	.39
NIV					
PEEP, mbar	8 (7-10)	8 (8-10)	8 (8-10)	8 (5.5-10)	.46
Pressure support, mbar	5 (5-8)	5 (4-7)	5 (5-8)	5 (5-8)	.44
Fio_2_, %	80 (70-90)	80 (70-100)	80 (80-85)	80 (60-85)	.26
IMV					
Pressure control methods	125 (25.1)	8 (5.8)	81 (32.8)	36 (31.3)	.77
Volume control methods	29 (5.8)	1 (0.7)	19 (7.7)	9 (7.8)	.83
PS mode	2 (0.4)	0	1 (0.4)	1 (0.9)	.79
Unknown method	24 (4.8)	3 (2.2)	6 (2.4)	15 (13)	.21
PEEP, mbar	8 (7-10)	9 (8-9)	8 (8-10)	8 (5-10)	.84
Tidal volume, mL	450 (420-510)	421 (348-450)	459 (422-519)	450 (421-500)	.041^a^
Tidal volume/kg body weight, mL/kg	5.8 (5.1-6.8)	5.6 (4.9-6.8)	5.8 (5.2-6.9)	6 (4.5-6.7)	.55
Fio_2_, %	100 (100-100)	100 (98-100)	100 (100-100)	100 (100-100)	.17
Duration, d	4 (2-6)	4.5 (3.3-6.5)	4 (2-6)	4.5 (1.8-6.3)	.85

Data are presented as No. (%) or median (interquartile range), unless otherwise indicated. HFNO = high-flow nasal oxygen; IMV = invasive mechanical ventilation; NIV = noninvasive ventilation; PEEP = positive end expiratory pressure; PS = pressure support.

a Significant difference among patients with mild, moderate, or severe ARDS.

**Figure 3:**
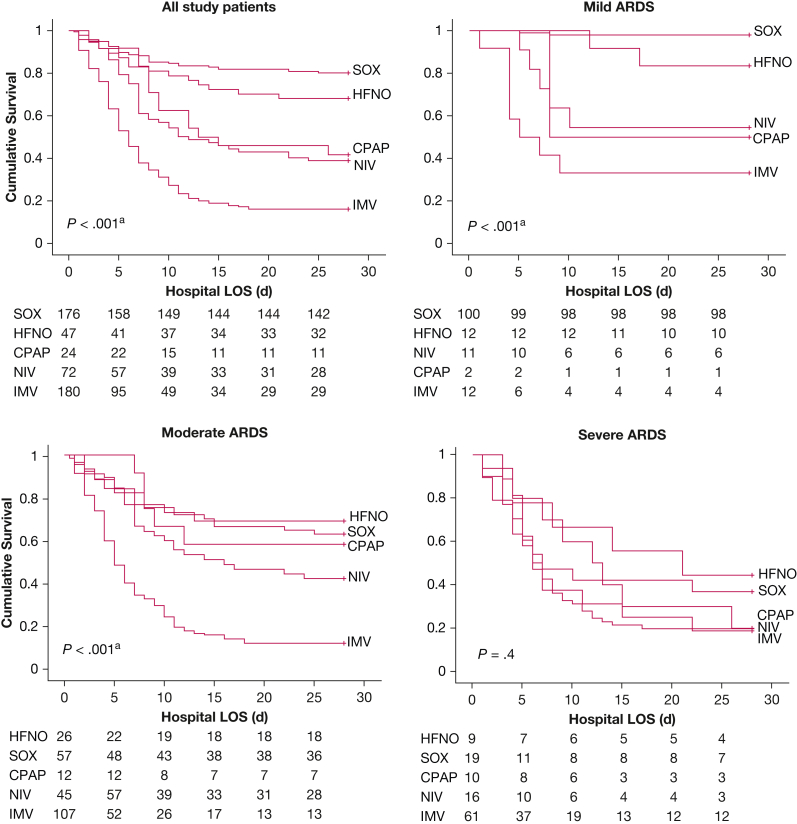
Kaplan-Meier curves showing survival by respiratory strategy. HFNO = high-flow nasal oxygen; IMV = invasive mechanical ventilation; LOS = legnth of stay; NIV = noninvasive ventilation; SOX = standard oxygen therapy.

The 28-day mortality rates associated with the use of different respiratory support techniques were as follows: SOX, 19.9% (35/176); HFNO, 31.9% (15/47); CPAP, 58.3% (14/24); NIV, 44/72 (61.1%), and IMV, 83.9% (151/180). Mortality did not differ significantly by category of facility (e-Table 2). Cumulative 28-day survival rates differed among the most advanced respiratory support techniques used in study patients categorized as having mild or moderate ARDS at hospital admission, but not in those with severe ARDS (Fig 3). Patients who required escalation of the first-line respiratory support technique showed an increased 28-day mortality when compared with patients who did not (81.8% [81/99] vs 44.5% [178/400]; *P < .*001).

**Figure 4:**
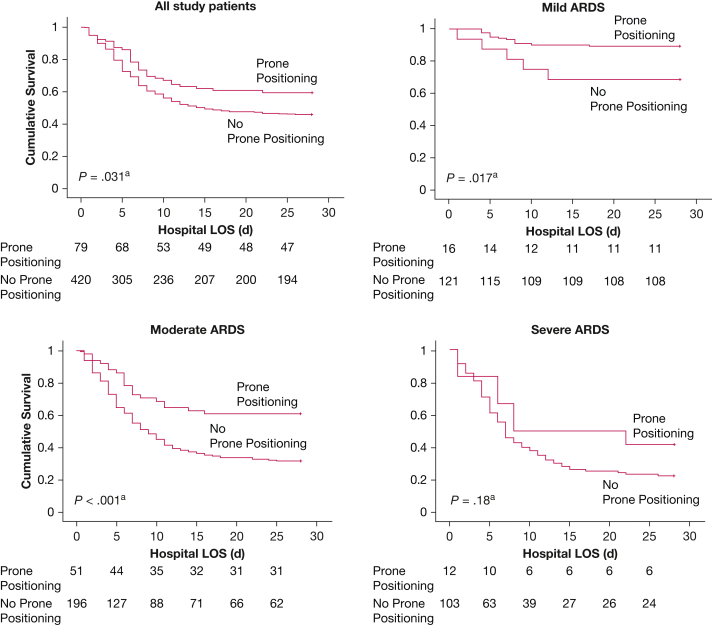
Kaplan-Meier curves showing survival by proning strategy. LOS = length of stay.

Seventy-nine study participants (15.8%) underwent prone positioning (mild ARDS, 11.7%; moderate ARDS, 20.6%; severe ARDS, 10.4%; *P = .*014); most of them were awake (74.7% [59/79]). Study participants who underwent prone positioning at any point during hospital admission showed a lower 28-day mortality (40.5% [32/79]) than patient who did not during the hospital stay (54% [227/420]; *P = .*027). Although patients with mild ARDS who underwent prone positioning showed higher 28-day mortality than patients who did not, 28-day mortality was lower in patients with moderate ARDS who were managed with prone positioning compared with those who were not (Fig 4).

The SpO_2_ to Fio_2_ ratio and respiratory rate at hospital admission, as well as the use of NIV or IMV as the most advanced respiratory support technique, were independent risk factors for 28-day mortality. No ventilator setting was associated independently with 28-day mortality in patients requiring IMV (Table 4).

**Table 4:** Risk Factors for In-Hospital Mortality

Variable	OR	95% CI	*P* Value
Risk factors for 28-d mortality			
Age, y	1.02	0.99-1.04	.11
Chronic arterial hypertension, binary	1	0.47-2.15	.99
SpO_2_ to Fio_2_ ratio at admission	0.99	0.98-0.99	< .001^a^
Respiratory rate at admission, breaths/min	1.07	1.03-1.12	.002^a^
Platelet count at admission, G/L	0.99	0.99-1	.11
Prone positioning, binary	0.5	1.2-1.29	.15
Most advanced respiratory support, binary			
SOX	Reference		
HFNO	0.37	0.07-1.86	.23
CPAP	2	0.55-7.04	.3
NIV	6.31	2.29-17.37	< .001^a^
IMV	8.08	3.51-18.57	< .001^a^
IMV ventilator settings and association with 28-d mortality			
Ventilation mode, binary			
Pressure controlled mode	Reference		
Volume controlled mode	0.58	0.12-1.72	.25
Fio_2_, %	1.06	0.97-1.15	.21
Tidal volume/kg body weight, mL/kg	0.81	0.52-1.26	.34
PEEP, mbar	0.93	0.82-1.06	.27

HFNO = high-flow nasal oxygen; IMV = invasive mechanical ventilation; NIV = noninvasive ventilation; PEEP = positive end expiratory pressure; SOX = standard oxygen therapy; SpO_2_ = plethysmographic oxygen saturation.

a Significant association with 28-day mortality.

## Discussion

This multicenter prospective observational explorative cohort study is to the best of our knowledge the first of its kind conducted in sub-Saharan Africa to report on the use and clinical outcomes of respiratory support techniques in patients with COVID-19-associated ARDS. We included 499 patients who were admitted to 13 COVID-19 referral hospitals in Uganda. The study was conducted during a 12-month period that included the first major COVID-19 surge in Uganda (September 2020 through February 2021). The demographic and clinical characteristics of the study population closely resemble those reported by the recent African COVID-19 Critical Care Outcomes Study (ACCCOS). Compared with critically ill populations without COVID-19 in sub-Saharan Africa,^7^ the median patient age of 54 years in the present cohort was high, but lower than that reported by studies from other continents.^15^^,^^16^ Similarly, the rate of premorbid conditions in the present cohort was higher than that observed in previous critically ill patient populations from Uganda,^13^ but still lower than the rate observed in patients with COVID-19 treated in high-income countries.^17^^,^^18^ In agreement with the ACCCOS, the patients included in our analysis demonstrated a high disease severity, as assessed by pulmonary and extrapulmonary organ dysfunction. In contrast to the ACCCOS, which included only patients with COVID-19 admitted to HDUs or ICUs in Africa, we included critically ill patients with COVID-19-associated ARDS, independent of whether they were treated on an ICU. Unsurprisingly, the rate of ICU admission in the present population was low: only 39% of patients were admitted to an ICU or HDU. Although we did not document the reasons why patients were not admitted to an ICU, it is highly likely that the lack of critical care capacity (note that only eight of 13 study centers had an ICU) played a role.

Thirty-five percent of the study patients were treated with SOX alone, 36.1% received invasive ventilation, and the remaining 28.6% of study patients received either NIV, CPAP, or HFNO therapy as the most advanced respiratory support technique. With some variations in the frequency of CPAP and HFNO use, these results are in keeping with the findings of the ACCCOS. In patients with severe ARDS, this ratio shifted toward advanced respiratory support techniques, namely IMV being applied as the most advanced respiratory support technique in 53% of the study patients. However, in comparison with high-income settings, the rate of IMV in patients with severe ARDS remains extremely low. Similarly, the failure rate of noninvasive respiratory support techniques in the present cohort was approximately 20%, and surprisingly low compared with international data.^18^ Notably, because almost without exception only patients requiring IMV were admitted to an ICU or HDU (180/194), most of the noninvasive respiratory support was provided in non-ICU settings. Although reports from high-income countries have indicated that such an approach may be feasible,^18^ our observations and the low rate of IMV provided to patients with (severe) COVID-19-associated ARDS underline the critical shortage of ICU capacity in Uganda.^19^

When analyzing our results regarding the settings used during noninvasive respiratory support, it is interesting that for CPAP and NIV, these did not differ significantly among patients with mild, moderate, or severe ARDS. Pressure control modes were used in more than two-thirds of patients who received invasive ventilation. In accordance with current guidelines,^4^^,^^6^ the results suggest that lung protective ventilation was applied widely in most study participants receiving IMV. In comparison with reports from high-income countries, the positive end expiratory pressure levels applied during IMV in our cohort (7-10 cm H_2_O) were low. The fact that continuous pulse oximetry monitoring was not available for all ICU beds at all study sites may explain the median Fio_2_ values of 100% across all ARDS severity strata.^6^

The 28-day mortality observed in this population with COVID-19-associated ARDS was high,^20^ compared with that reported from Europe or North America.^15^^,^^21^ In addition, although the studies that reported on the outcomes of COVID-19 in Uganda did not specifically document (clinical or treatment) respiratory characteristics of patients, the mortality rate among critically ill patients (who often had ARDS) was comparable with the results that we obtained.^22^, ^23^, ^24^ Once again, when comparing mortality rates between studies and settings, it is important to remember that nearly two-thirds of this study population was treated in a non-ICU setting, despite receiving advanced respiratory support in many instances. Indeed, a study looking at the prevalence and outcomes of non-COVID-19 ARDS in Ugandan ICUs reported lower mortality rates among mechanically ventilated patients with ARDS.^25^ In line with the ACCCOS, we identified the severity of respiratory derangement and positive pressure mechanical ventilation (NIV and IMV) as independent risk factors for death resulting from COVID-19-associated ARDS.

Although mortality rates were highest in patients receiving NIV or IMV as the most advanced respiratory support, it was lowest in patients who were treated with SOX or HFNO therapy. These differences were significant for mild and moderate ARDS, but not for severe ARDS. The multiple logistic regression analysis suggested that this association may be explained partly by disease severity, but also highlighted an independent association between mechanical ventilation, particularly IMV, and mortality. The fact that patients with COVID-19-associated ARDS receiving IMV were sedated routinely and usually were ventilated in controlled methods made them exquisitely vulnerable to power cuts and interruptions of pressurized oxygen supply, both of which occurred on multiple occasions during the study period. In addition, limited human and material resources in non-ICU and ICU wards may have contributed to the very high mortality rates we observed.

Another interesting finding of this study is that patients with COVID-19-associated ARDS who underwent prone positioning showed a lower 28-day mortality than patients who did not. The mortality difference was particularly pronounced in patients who underwent awake prone positioning and in those with moderate, but not mild or severe, ARDS. These results are in agreement with published data suggesting improved oxygenation, lower intubation rates, and a survival benefit for awake prone positioning in COVID-19-associated ARDS,^26^ particularly when performed early^27^ and in patients with moderate ARDS.^28^

When interpreting the results of our study, certain limitations need to be considered. Although the number of patients included was high (n = 499), no formal power analysis could be undertaken at the time when the study was designed. It is unlikely that this has affected the power of our study to report its primary study objective. However, we cannot exclude that our study was underpowered to address some study objectives with a sufficient reliability. This may be particularly relevant when interpreting comparisons between patients with different ARDS severity strata. In addition, because of the observational design of this study, we only could report observations and associations and could not demonstrate any causative relationships. Therefore, the results of our study highlighting differences in mortality rates among respiratory support techniques should be considered as hypothesis generating. Finally, the care of critically ill patients in sub-Saharan Africa is profoundly challenged by human, material, and logistical limitations. We did not systematically document the influence of such restrictions, and therefore cannot determine their influence on the respiratory support technique provided to and clinical outcomes of patients with COVID-19-associated ARDS.

In conclusion, SOX, HFNO, CPAP, NIV, and IMV were used as respiratory support techniques in patients with COVID-19-associated ARDS in Uganda. Two-thirds of study participants were cared for outside ICU settings, underlining the lack of ICU capacity in the country. The 28-day all-cause mortality of this cohort was high, particularly in patients with moderate or severe COVID-19-assosciated ARDS, as well as in those receiving NIV or IMV. Although our data are observational, they suggest that the use of SOX and HFNO therapy as well as awake prone positioning are associated with a lower mortality resulting from COVID-19-associated ARDS in a resource-limited setting.

## Interpretation

SOX, HFNO, CPAP, NIV, and IMV were used as respiratory support techniques in patients with COVID-19-associated ARDS in Uganda. ICU capacity was very limited and resource constraints were severe. The need for escalation to advanced respiratory support (especially mechanical ventilation) was associated with high mortality in this population.

## Funding/Support

A. K. was supported through the Developing Excellence in Leadership, Training, and Science in Africa [Grant DEL-15-011] to Training Health Researchers into Vocational Excellence 2 program. The DELTAS Africa Initiative is an independent funding scheme of the African Academy of Science’s 10.13039/501100014163Alliance for Accelerating Excellence in Science in Africa and is supported by the New Partnership for Africa’s Development Planning and Coordinating Agency, with funding from the 10.13039/100010269Wellcome Trust [grant 107742/Z/15/Z] and the UK government. The study also was supported by the Makerere Research and Innovation Fund.

## Financial/Nonfinancial Disclosures

None declared.

## Supplementary Data

e-Online Data

e-Figure
