# Supplementary material for: Respiratory Support Techniques for COVID-19-Related ARDS in a Sub-Saharan African Country: A Multicenter Observational Study
Source: Chest. 2023 Feb 10;164(2):369–80. doi: 10.1016/j.chest.2023.01.039 (PMC9911971; doi:10.1016/j.chest.2023.01.039)
Supplement: e-Online Data [file mmc1.docx]

**e-Table 1: Respiratory strategy by facility and facility category**

|  | | Respiratory Strategy | | | | |  |
| --- | --- | --- | --- | --- | --- | --- | --- |
| Facility | Category | SOX | HFNC | CPAP | NIV | IMV | Total |
| Mbarara regional referral hospital | Public | 22 | 3 | 4 | 20 | 11 | 60 |
| Masaka regional referral hospital | Public | 13 | 2 | 3 | 5 | 4 | 27 |
| TMR | Private | 8 | 7 | 0 | 0 | 12 | 27 |
| Kiruddu national referral hospital | Public | 19 | 3 | 5 | 0 | 0 | 27 |
| Rubaga Hospital | *Private Not for Profit | 12 | 2 | 4 | 3 | 2 | 23 |
| Mengo Hospital | *Private Not for Profit | 11 | 5 | 8 | 25 | 8 | 57 |
| St. Mary’s Hospital, Lacor | *Private Not for Profit | 18 | 4 | 7 | 15 | 8 | 52 |
| Mbale regional referral hospital | Public | 21 | 1 | 3 | 3 | 3 | 31 |
| Jinja regional referral hospital | Public | 19 | 1 | 2 | 16 | 7 | 45 |
| Kabale regional referral hospital | Public | 9 | 2 | 3 | 5 | 7 | 26 |
| Kampala hospital | Private | 8 | 9 | 10 | 11 | 15 | 53 |
| St Francis Hospital, Nsambya | *Private Not for Profit | 11 | 3 | 6 | 10 | 11 | 41 |
| Entebbe regional referral hospital | Public | 15 | 8 | 3 | 4 | 0 | 32 |

- Private Not For Profit, Missionary/Faith based hospitals generally more affordable than Private for profit hospitals.

**e-Table 2: Mortality stratified by facility category and respiratory strategy at admission**

|  | Proning  n(%) | SOX | Mortality n (%) | HFNC | Mortality n (%) | CPAP | Mortality n (%) | NIV | Mortality n (%) | IMV | Mortality n (%) | Total Mortality N (%) |
| --- | --- | --- | --- | --- | --- | --- | --- | --- | --- | --- | --- | --- |
| Public hospitals | 41(16.1) | 118 | 28(24) | 20 | 10(50) | 23 | 19(83) | 59 | 49(83) | 34 | 30(88) | 136 (53.5) |
| Private hospitals | 38(15.5) | 68 | 17 (25) | 30 | 8(27) | 35 | 16(47) | 58 | 43(74) | 54 | 40(74) | 124(50.6) |
