# Supplementary figures and images for: Respiratory Support Techniques for COVID-19-Related ARDS in a Sub-Saharan African Country: A Multicenter Observational Study
Source: Chest. 2023 Feb 10;164(2):369–80. doi: 10.1016/j.chest.2023.01.039 (PMC9911971; doi:10.1016/j.chest.2023.01.039)

eFigure 1. Map of Uganda showing distribution of study hospitals

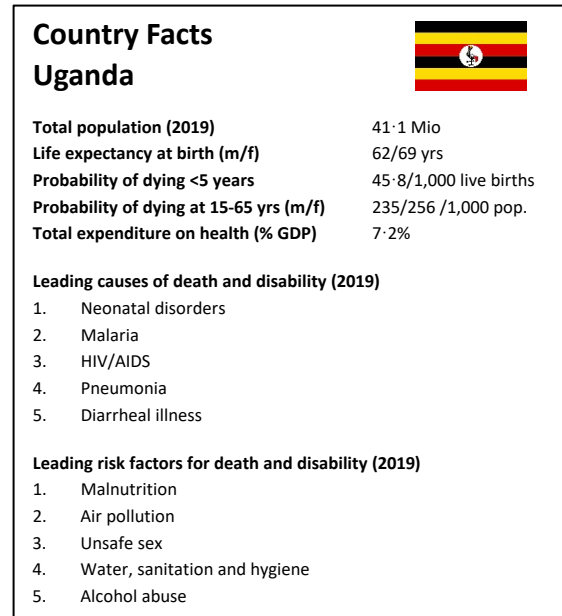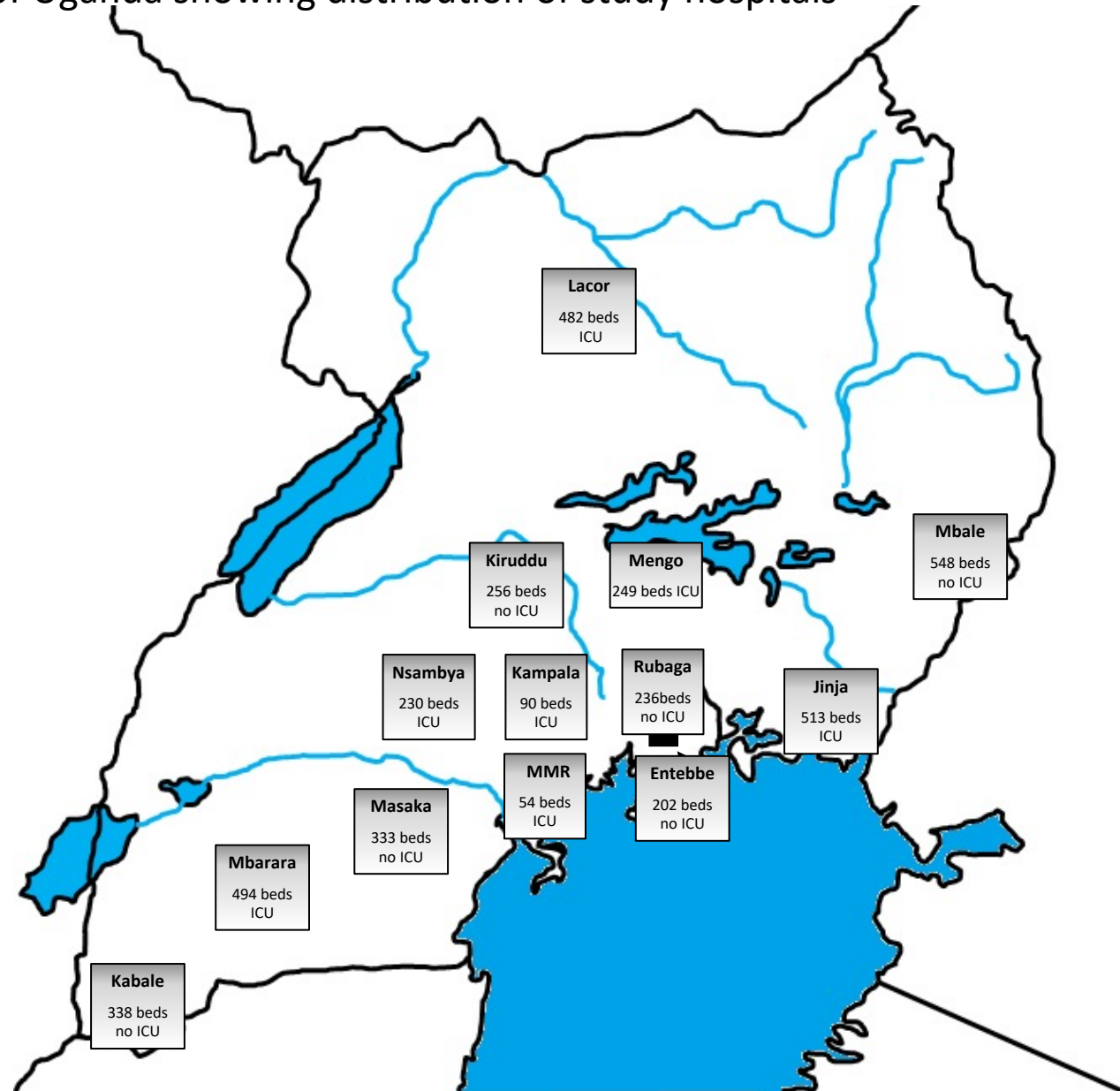

Supplement: e-Figure [file mmc2.pdf]
